# ROA: A Rapid Learning Scheme for *In-Situ* Memristor Networks

**DOI:** 10.3389/frai.2021.692065

**Published:** 2021-10-15

**Authors:** Wenli Zhang, Yaoyuan Wang, Xinglong Ji, Yujie Wu, Rong Zhao

**Affiliations:** Department of Precision Instrument, Center for Brain Inspired Computing Research, Beijing Innovation Center for Future Chip, Tsinghua University, Beijing, China

**Keywords:** memristor, meta-learning, fast adaptation, few-shot learning, neuromorphic

## Abstract

Memristors show great promise in neuromorphic computing owing to their high-density integration, fast computing and low-energy consumption. However, the non-ideal update of synaptic weight in memristor devices, including nonlinearity, asymmetry and device variation, still poses challenges to the *in-situ* learning of memristors, thereby limiting their broad applications. Although the existing offline learning schemes can avoid this problem by transferring the weight optimization process into cloud, it is difficult to adapt to unseen tasks and uncertain environments. Here, we propose a bi-level meta-learning scheme that can alleviate the non-ideal update problem, and achieve fast adaptation and high accuracy, named Rapid One-step Adaption (ROA). By introducing a special regularization constraint and a dynamic learning rate strategy for *in-situ* learning, the ROA method effectively combines offline pre-training and online rapid one-step adaption. Furthermore, we implemented it on memristor-based neural networks to solve few-shot learning tasks, proving its superiority over the pure offline and online schemes under noisy conditions. This method can solve *in-situ* learning in non-ideal memristor networks, providing potential applications of on-chip neuromorphic learning and edge computing.

## Introduction

Memristors are considered as leading device candidates for neural network accelerators (Yang et al., 2013; Chen et al., 2015; Tsai et al., 2018; Zidan et al., 2018) due to their ability to physically store synaptic weights in conductance state, which enable in-memory computing. Implementation of neural networks in memristor-based hardware exhibits high density integration, low power consumption and high efficiency (Cai et al., 2019; Yu 2018). It can also greatly promote the development of brain-inspired computing systems to achieve human-like intelligence (Zhang et al., 2016). However, memristors possess some non-ideal properties that challenge the hardware implementations. The weight updates on memristors are asymmetric, nonlinear and low precision, significantly degrading the learning accuracy (Kataeva et al., 2015; Agarwal et al., 2016; Wang et al., 2020a). Additionally, the outputs of networks, determined by input currents and conductance of memristors, are also perturbed by the variability of circuits, including input currents, reference voltage, output resistance (Yang et al., 2013; Agarwal et al., 2016).

Currently, there are mainly two types of co-optimization learning schemes to overcome these challenges (Hu et al., 2016; Zidan et al., 2018; Agarwal et al., 2016; Chen et al., 2015). One type is online learning, which allows training models to be implemented on neuromorphic hardware by using backpropagation (Yu et al., 2016) or biological local learning, such as spike-timing-dependent plasticity (STDP) (Guo et al., 2017). For fast online learning, conductance tuning with less operations on hardware is preferred, which the weights of networks are directly written without verification by reading. However, the nonlinearity and asymmetry of memristors cause the accuracy loss of the neural networks during learning (Yu et al., 2016; Kataeva et al., 2015). To mitigate the adverse effects of memristors, various approaches have been reported. Some work initialized the weights at each update step to achieve linear and symmetric weights (Li et al., 2019; Geminiani et al., 2018). Retraining of networks and developing highly robust algorithms, such as Neural State Machine, have also been proved to overcome the asymmetric properties of memristors to a certain degree (Liu et al., 2017; Tian et al., 2020; Tian et al., 2021). The other type is offline learning, which maps the pre-trained network to hardware, and only performs inference in neuromorphic chips (Hu et al., 2016; Shafiee et al., 2016; Chi et al., 2016). The non-ideality of weight updates can be concealed by iterative programming with a write-verify technique, reading the conductance and rewriting for accuracy. However, for a new task, the entire process must be restarted from scratch through offline learning. For most algorithms, all tunable parameters in the neural network must be re-trained for a new task, resulting in a large number of operations. Therefore, there is usually a trade-off between speed and performance for memristor networks from offline learning to online learning.

Different from the current on-chip learning schemes, humans can quickly adapt to the environment by drawing on prior experience or learning to learn. In machine learning, this learning approach is named meta-learning (Thrun and Pratt 1998), which has made significant progress in recent years (Wang et al., 2020b; Hospedales et al., 2020; Vanschoren, 2018). There are many meta-learning techniques, such as optimizee (Andrychowicz et al., 2016; Ravi and Larochelle 2017), metric based (Hu et al., 2020; Vinyals et al., 2016; Snell et al., 2017) and fine-tuning (Antoniou et al., 2018; Li et al., 2017; Finn et al., 2017; Nichol et al., 2018). Particularly, Model-Agnostic Meta-Learning (MAML) (Finn et al., 2017) is a general meta-learning framework that provides a good initial condition of network for fine-tuning on similar tasks, which can simplify the optimization to a few steps for new unseen tasks. MAML can also be applied to fields such as reinforcement learning (Gupta et al., 2018) and continual learning (Al-Shedivat et al., 2018). The studies on the silicon-based neuromorphic chips have proven that meta-learning schemes can significantly accelerate the learning of new tasks and improve their performance (Bohnstingl et al., 2019; Stewart et al., 2020). However, optimization methods for memristor-based networks with the meta-learning scheme have yet to be developed.

**FIGURE 1:**
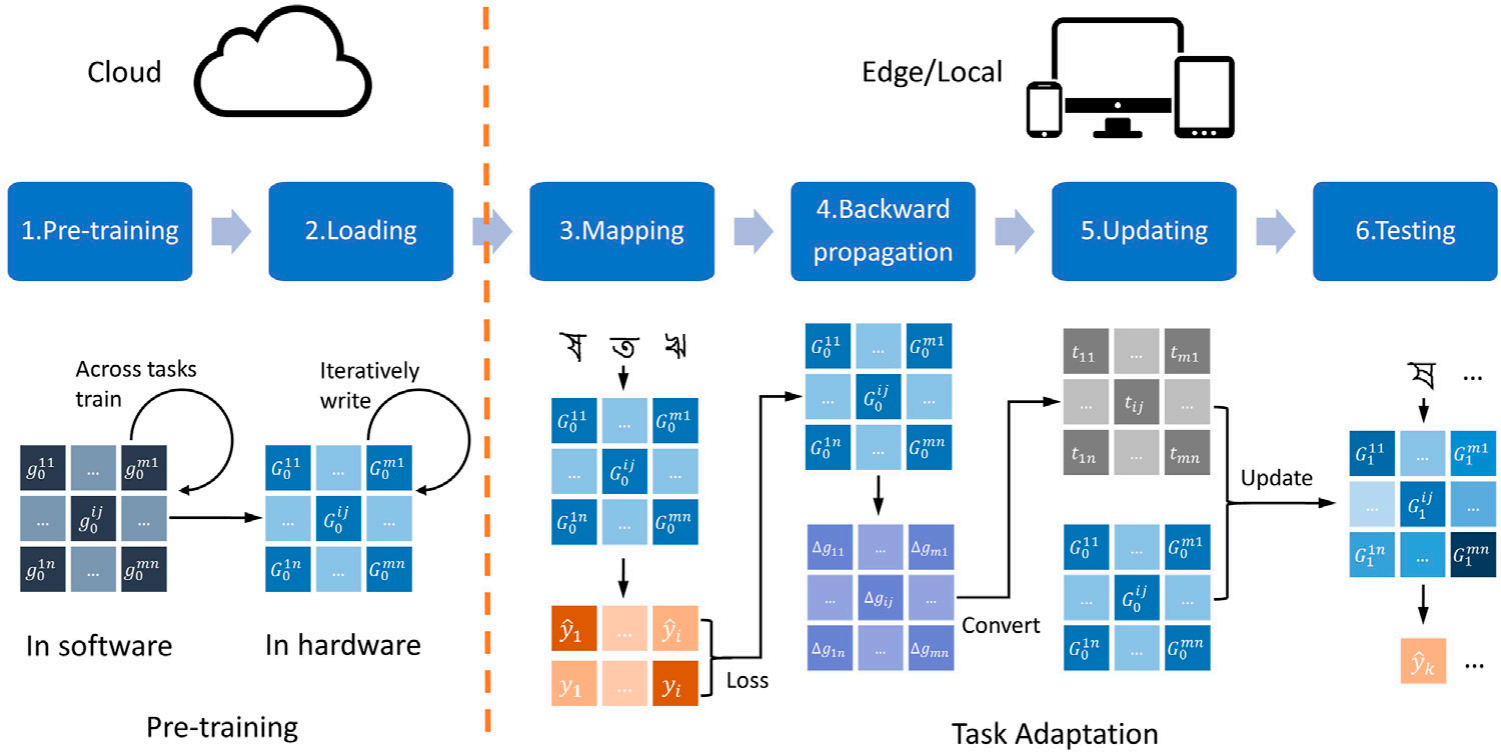
The framework of ROA (Rapid One-step Adaption). In the pre-training phase: (1) we train a good initial network across tasks with parameters $g_{0}^{ij}$ ; (2) we load it into the memristor array as $G_{0}^{ij}$ with iterative programming with write-verify. In the task adaptation phase: (3) we map the task into voltage input of the memristor for prediction ${\hat{y}}_{i}$ , then we calculate the loss function with true label $y_{i}$ ; (4) we calculate the gradient descent $\text{Δ}g_{ij}$ with loss by backpropagation; (5) we convert $\text{Δ}g_{ij}$ into update pulse period ${\text{t}}_{ij}$ and update the hardware network; (6) we evaluate the network on the testing dataset. The color and its brightness of the matrix indicate different forms of data and its value.

In this work, we propose a meta-learning scheme for memristor-based neural networks that can overcome the non-ideal synapse weights for training and provide improved performance. Our method consists of two phases, including pre-training and task adaptation, as shown in Figure 1. Firstly, a good initial network for a group of tasks is trained in software and then mapped to hardware by iterative programming with write-verify. Then, a rapid training in one-step adaption is performed for an unseen task with a few samples of the *in-situ* hardware network. This scheme can free the memristor networks from unnecessary operations, mitigating the problem of performance degradation in online learning. It also has the ability to accomplish new tasks through quick adaptations, which is more powerful than the offline trained networks. Since only one update step is needed, a new task requires significantly less training time, only a few samples and little computation consumption. These merits make our scheme very suitable for situations with limited computing power and limited data, such as edge computing. Our main contributions are as follows:

1. We propose a hybrid learning scheme of offline learning and online learning for meta-learning on memristor-based neural networks. It combines the advantages of offline learning and online learning for the hardware to achieve high accuracy and fast adaption for unseen tasks.
2. We report the Rapid One-step Adaption (ROA) algorithm, which enables memristor-based neural networks with meta-learning capability. It mitigates the effects of non-ideal characteristics of memristor-based neural networks, and achieves superior performance by introducing dynamic learning rate, regularization constraint and one-step adaption.
3. In order to evaluate our model on few-shot tasks, we built a simulator based on the experimental characteristics of memristor, which can better support the acceleration of large-scale network and the quantitative analysis of networks with noise. On this basis, we comprehensively evaluate the proposed model on two typical few-shot learning datasets. Our results reveal a good performance of memristor networks on few-shot learning task with significant improvement of accuracy than the baseline.

## Experimental Setup

In this section, we discuss the setup of memristors for the simulation experiment. In *Memristor Model*, we introduce the model of memristors for simulation based on our experimental data and discuss the properties of memristors in this work. In *Simulator for Memristor Networks*, we introduce the simulator for this work, including weight update, noise setting and mapping.

### Memristor Model

In this work, we implemented a two-terminal ${\text{TaO}}_{\text{x}}/{\text{HfO}}_{2}/\text{Ta}$ bi-layered resistive switching device (Kim et al., 2018) as a synaptic device. The electrical conductance of a memristor is generally determined by the conductive filaments, which are formed and ruptured due to the electromigration of oxygen vacancies under an electric field. We adopted the memristor model reported in Wang et al. (2020b), which uses a state parameter ω∈[0, 1] to describe the area covered by the filaments in memristors. The dynamic change of  ω in response to the external voltage V yields the following relationships (Choi et al., 2015):

$$\begin{array}{c} \frac{d\omega }{dt}=\{\begin{array}{c} {\left(1-\omega \right)}^{2}k\left(e^{-\mu_{1}V}-e^{\mu_{2}V}\right),V<0,\\ \omega^{2}k\left(e^{-\mu_{1}V}-e^{\mu_{2}V}\right),V>0. \end{array} \end{array}$$ (1)

where k , $\mu_{1}$ , $\mu_{2}$ are positive parameters determined by the material properties, k is the ion hopping distance, and $\mu_{1}$ and $\mu_{2}$ are the hopping barrier heights. In Eq. 1, the change of ω , dω/dt , depends on the exponentially dependent dynamics of voltage V . Thus, the states of ω will only be slightly disturbed when pulse voltage is low (such as below 0.1 V). This allows the memristor network to work normally under reading pulses and maintain consistent weights. The current I through the memristor is determined by Choi et al. (2015):

$$\begin{array}{c} I=\omega \gamma sinh\left(\delta V\right)+\left(1-\omega \right)\alpha \left(1-e^{-\beta V}\right),\; \end{array}$$ (2)

where γ is the effective tunneling distance, δ is the tunneling barrier, α is the depletion width of the Schottky barrier region, and β is the Schottky barrier height. They are all positive parameters determined by materials.

**FIGURE 2:**
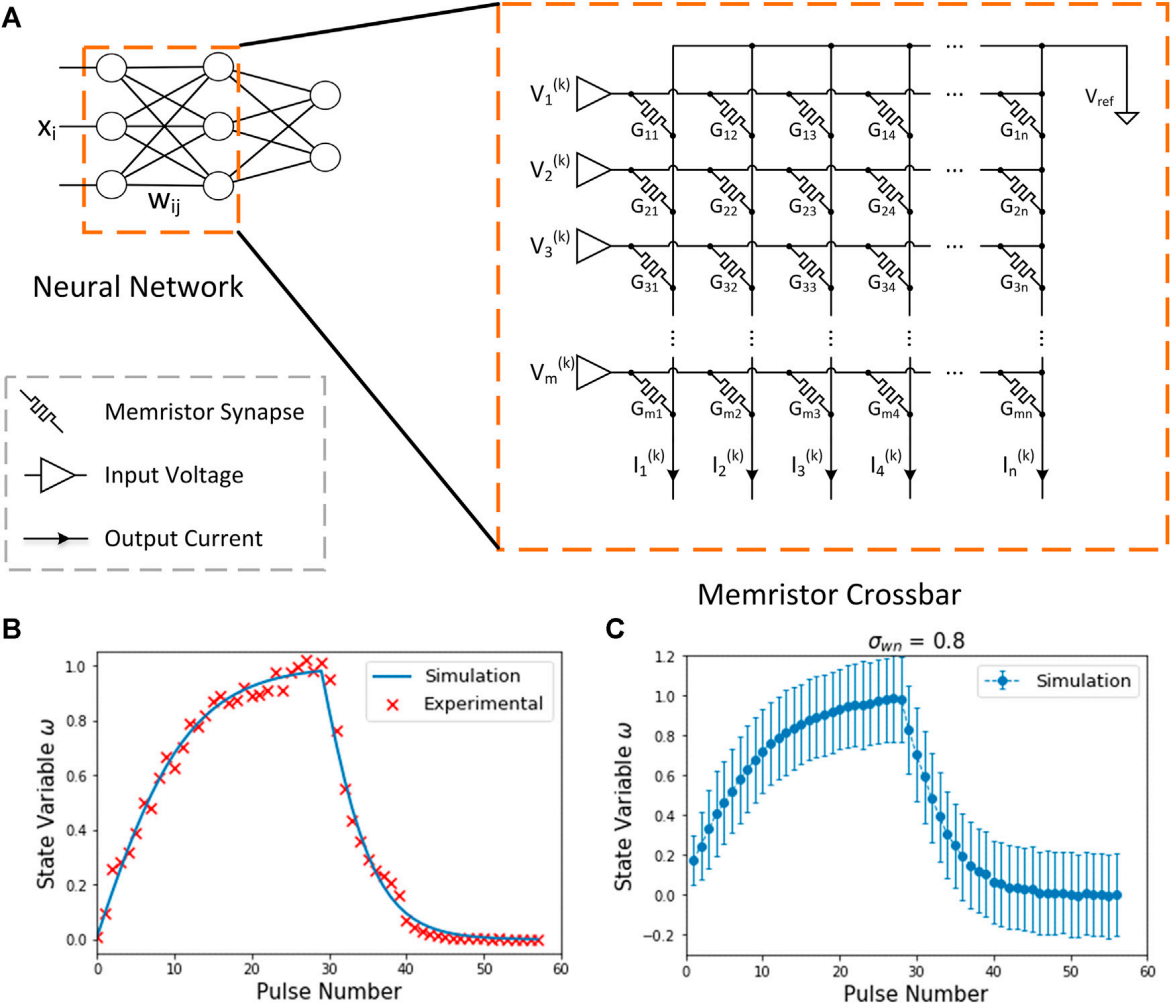
**(A)** Memristor Crossbar. The weights of neural network are mapped to memristor crossbar arrays as conductance $G_{ij}$ . The inputs and outputs are represented by the voltages $V_{i}$ and the currents $I_{i}$ . **(B)** Experimental and simulated updates of ${\text{TaO}}_{\text{x}}/{\text{HfO}}_{2}/\text{Ta}$ bi-layered memristors. **(C)** Standard variation of simulated updates with write noise.

In the measurement of the device, we applied negative pulses in increasing amplitudes from −0.8 to −3.8 V (in 0.1 V increments) and 100 µs width for the potentiation process, and positive pulses in decreasing amplitudes from 3.5 to 0.8 V (in 0.1 V decrements) and 100 µs width for the depression process. A model of the memristor behavior was built based on the experimental data. As shown in Figure 2B, the simulation results of the memristor are in good agreement with the experimental data, indicating that the model can properly reproduce the behavior of the device.

Memristors have a strong non-linear conductance. Particularly, the conductance change is more subtle when the conductance state is approaching its maximum or minimum value. Memristors also have an asymmetric conductance change behavior. The potentiation and depression pulses cause the conductance to change with different magnitudes depending on the direction of change. On the other hand, the variation in memristor devices is inherently a stochastic process due to the movement of atoms or oxygen vacancies. This process is interfered by various noises, and usually modeled using normal distribution (Agarwal et al., 2016). In addition, the updated states of memristor synapses are discrete because of the limited conductance levels and discrete programming pulse width. All these features will be considered in our simulation.

### Simulator for Memristor Networks

We built a simulator using Pytorch (Paszke et al., 2019) for the simulation of training and inference processes, and further quantitatively analyzed the effects of the non-ideal characteristics of the device. The simulator supports the GPU acceleration for large-scale network in Pytorch. The pulses of potentiation and depression were set with fixed amplitude and duration. The parameters of the memristor are shown in Table 1.

**TABLE 1:** List of the parameters used in the memristor model.

Parameter	Value	Parameter	Value	Parameter	Value
k	1e-4	δ	0.5	${\text{V}}_{\text{p}}$	−1.1 V
${\text{μ}}_{1}$	19.25	α	1.58e-3	${\text{T}}_{\text{p}}$	3 µs
${\text{μ}}_{2}$	13	β	0.5	${\text{V}}_{\text{d}}$	1.4 V
γ	3.01e-3	${\text{V}}_{\text{r}}$	0.05 V	${\text{T}}_{\text{d}}$	30 µs

Normally, a crossbar structure is adopted in memristor networks, as shown in Figure 2A. The conductance state of a memristor represents the synaptic value in neural networks. The inputs are mapped to the input voltages and the outputs are represented by currents. In a crossbar structure, the transmitted signal is determined by the product of input signals and synaptic weights through Ohm’s law and Kirchhoff’s law. Thus, multiply-accumulations (MACs) can be physically performed at the weight locations, greatly reducing computing operations and energy consumption. For multilayer networks, the output currents of the previous layer can be converted to voltage for the input of the next layer. Each layer follows the configuration in the inference process until the final layer.

According to Eq. 2, the relation between voltage and current of a memristor is approximately linear when voltage is low (such as below 0.1 V). Then the conductance of a synapse in the crossbar can be read with read voltage $V_{r}$ by:

$$\begin{array}{c} G_{ij}=\frac{\left[\omega_{ij}\gamma \text{sinh}\left(\delta V_{r}\right)+\left(1-\omega_{ij}\right)\alpha \left(1-e^{-\beta V_{r}}\right)\right]}{V_{r}}. \end{array}$$ (3)

The state $\omega_{ij}$ of the memristor yields

$$\begin{array}{c} \omega_{ij}=\frac{G_{ij}V_{r}-\alpha \left(1-e^{-\beta V_{r}}\right)}{\gamma \text{sinh}\left(\text{δ}V_{r}\right)-\alpha \left(1-e^{-\beta V_{r}}\right)}. \end{array}$$ (4)

During the updating process, the expected changing state $\text{Δ}\omega_{ij}$ of the memristor yields

$$\begin{array}{c} \text{Δ}\omega_{ij}=\frac{\text{Δ}G_{ij}V_{r}}{\gamma \text{sinh}\left(\text{δ}V_{r}\right)-\alpha \left(1-e^{-\beta V_{r}}\right)}. \end{array}$$ (5)

The (potentiation/depression) update pulses are defined as identical with fixed amplitudes ( ${\text{V}}_{\text{p}}/{\text{V}}_{\text{d}}$ ) and widths $\left({\text{T}}_{\text{p}}/{\text{T}}_{\text{d}}\right)$ in the programming process. Thus, the programming pulse number $n_{ij}$ should be rounded to an integer. The time of the programming pules, $t_{ij}$ , can be obtained as

$$\begin{array}{c} \begin{array}{c} t_{ij}=n_{ij}\cdot T_{p/d}=\left[\frac{\text{Δ}\omega_{ij}}{\lambda k\left(e^{-\mu_{1}V_{p/d}}-e^{\mu_{2}V_{p/d}}\right)\left(\lambda -\text{Δ}\omega_{ij}\right)T_{p/d}}\right]\cdot T_{p/d}\;\\ \begin{array}{c} \lambda =\{\begin{array}{cc} \begin{array}{c} \left(1-\omega_{ij}\right) \end{array} & ,V=V_{p},\\ -\omega_{ij} & ,V=V_{d}. \end{array} \end{array} \end{array} \end{array}$$ (6)

To simulate the variation in real devices, we introduce write noise at each step of weight change following the work of Agarwal et al. (2016):

$$\begin{array}{c} \begin{array}{c} G=G_{0}+\text{Δ}G+N\left(\sigma \right)\\ \text{σ}=\sqrt{\Delta G\times \left({\bar{G}}_{max}-{\bar{G}}_{min}\right)}\times \sigma_{WN}\;, \end{array} \end{array}$$ (7)

where $G_{0}$ is the initial conductance of synapse, ΔG is the change of conductance, and N(σ) is a Gaussian distribution with standard variance σ . The variance of noise ${\text{σ}}^{2}$ is proportional to the range of conductance $\left({\bar{G}}_{max}-{\bar{G}}_{min}\right)$ and ΔG . $\sigma_{WN}$ is a dimensionless standard deviation. The standard deviation of the simulated conductance for $\sigma_{WN}=0.8$ is shown in Figure 2C.

The input voltage ${\text{V}}_{\text{in}}$ in Figure 2A is in the range [0, 0.1], which is low enough to stabilize the conductance states. The input vector $x_{in}$ is normalized to [0, 1]. Thus, the $x_{in}$ is mapped into voltage pulse ${\text{V}}_{\text{in}}$ by

$$\begin{array}{c} {\text{V}}_{\text{in}}=0.1x_{\text{in}}.\; \end{array}$$ (8)

The synaptic weights, $g_{\text{ij}}$ , of the neural network are mapped into the conductance values, $G_{\text{ij}}$ , of the memristor by:

$$\begin{array}{c} \begin{array}{c} g_{ij}=aG_{ij}-b\\ a=2/\left({\bar{G}}_{max}-{\bar{G}}_{min}\right)\\ b=\frac{{\bar{G}}_{max}-{\bar{G}}_{min}}{{\bar{G}}_{max}+{\bar{G}}_{min}}\\ {\bar{G}}_{max}=\frac{\gamma \text{sinh}\left(\text{δ}V_{r}\right)}{V_{r}}\\ {\bar{G}}_{min}=\frac{\alpha \left(1-e^{-\beta V_{r}}\right)}{V_{r}}, \end{array} \end{array}$$ (9)

where ${\bar{G}}_{max}$ and ${\bar{G}}_{min}$ are mean values of maximum and minimum conductance, respectively. Obviously, the value of $g_{ij}$ is limited in the range of [1,−1] .

The current collected at the output of each column j in the network array under the input voltage $V_{i}$ of each row i can be obtained as:

$$\begin{array}{c} \begin{array}{ccc} I_{j} & = & \underset{i}{\sum }\left[\omega_{ij}\gamma \text{sinh}\left(\delta V_{i}\right)+\left(1-\omega_{ij}\right)\alpha \left(1-e^{-\beta V_{i}}\right)\right]\\ & \approx & \underset{i}{\sum }V_{i}G_{ij}, \end{array} \end{array}$$ (10)

The output results $y_{j}$ of MAC should be mapped by the current:

$$\begin{array}{c} y_{j}=\sum_{i}g_{ij}x_{i}\approx 10\cdot aI_{j}-b\sum x_{in}\cdot \end{array}$$ (11)

In the configuration of the simulation, the initial weights ${\text{g}}_{\text{ij}}$ are implemented to the memristor networks through iterative programming to ensure high precision. Then, the weight update values $g_{ij}$ corresponding to $g_{ij}\;$ are calculated for each memristor through the error backpropagation algorithm. The programming pulse time $t_{ij}$ can be calculated by Eq. 6 and implemented to hardware after obtaining $g_{ij}$ . The variations are considered in these training steps and subsequent test processes.

## Methods

In this section, we introduce ROA, the proposed meta-learning algorithm for memristor neural networks, and MAML, a typical meta-learning algorithm, together with its derivatives that are the basis of our algorithm.

### Model-Agnostic Meta-Learning

Considering a supervised learning task T , it consists of training set $D_{\text{train}}=\left\{(x_{1}^{train},y_{1}^{train}\right),\left(x_{2}^{train},y_{2}^{train}\right),\ldots ,\left(x_{k}^{train},y_{k}^{train})\right\}$ and testing set $D_{\text{test}}=\left\{(x_{1}^{test},y_{1}^{test}\right),\left(x_{2}^{test},y_{2}^{test}\right),\ldots ,\left(x_{n}^{test},y_{n}^{test})\right\}$ , in (input image, output label) pairs. One solution to task T is to train model f with parameter θ by solving:

$$\begin{array}{c} \theta^{\text{*}}=\text{arg }\underset{\theta }{\min}\underset{i}{\sum }ℒ\left(y_{i}^{test},f_{\theta }\left(x_{i}^{test}\right),\eta \right)=\text{arg }\underset{\theta }{\min}ℒ\left(D_{test};\theta ,\eta \right), \end{array}$$ (12)

where  ℒ is the loss function that measures the distance between the prediction of $f_{\theta }$ and the true labels $\left\{y_{i}^{test}\right\}$ , η represents the choice of optimization of θ , which is usually stochastic gradient descent in neural networks. Consistently, we assume that $D^{\text{train}}$ and $D^{\text{test}}$ share the same distribution so that Eq. 12 can be approximately equivalent to:

$$\begin{array}{c} \theta^{*}=\text{arg }\underset{\theta }{\min}ℒ\left(D_{train};\theta ,\eta \right).\; \end{array}$$ (13)

Generalization power of the model is the key to the realization of this hypothesis.

In conventional deep learning, the optimization of each task $T_{i}$ starts from scratch, which requires hundreds of data samples and iterations. After optimization, a well-trained network is only effective for task $T_{i}$ corresponding to the distribution of the training data. In contrast, the goal of meta-learning is to train a model that can rapidly adapt to a new task $T_{i}$ using a small number of samples and a few training epochs. More specifically, it optimizes the meta-optimization method η using previous tasks, which significantly reduces the sample requirements and computation cost for the subsequent new tasks. The generalization power of meta-learning has been shifted from data distribution to tasks. For a distribution of tasks p(T) , the target of meta-learning can be expressed as:

$$\begin{array}{c} \eta^{\text{*}}=\text{arg }\underset{\eta }{\min}ℒ\left(p\left(T\right);\theta ,\eta \right). \end{array}$$ (14)

MAML (Finn et al., 2017) is a typical meta-learning framework for few-shot learning tasks, which can be rapidly updated for a few-shot task by learning good initialization parameters $\theta_{0}^{\text{*}}\;$ for a network. In other words, it optimizes the initial parameters $\theta_{0}$ of neural network as meta-optimizer η . In general, there are two phases of MAML: meta-testing and meta-training. In the meta-testing phase, it updates network weights θ  by stochastic gradient descent after i -step on training data from support task $S_{a}=\left\{D_{\text{train}}^{\left(a\right)},D_{\text{test}}^{\left(a\right)}\right\}$ , which can be expressed as:

$$\begin{array}{c} \theta_{i}^{\left(a\right)}=\theta_{\text{i}-1}^{\left(a\right)}-\alpha \nabla_{\text{θ}}L\left(D_{\text{train}}^{\left(a\right)},\theta_{i-1}^{\left(a\right)}\right), \end{array}$$ (15)

where α is the inner learning rate, and $\theta_{i}^{\left(a\right)}$ is the network weight after i -step towards task a . After n-step updating, the testing result can be displayed by testing dataset $D_{test}^{\left(a\right)}$ with parameters $\theta_{n}^{\left(a\right)}$ .

In the meta-training phase, it updates the initial parameters $\theta_{0}$ using the result in the meta-testing phase by gradient descent. The update for the meta-parameters $\theta_{0}$ can be expressed as:

$$\begin{array}{c} \theta_{0}^{\text{*}}=\theta_{0}-\beta \nabla_{\theta }\;\underset{a}{\sum }ℒ\left(D_{test}^{\left(a\right)},\theta_{n}^{\left(a\right)}\right), \end{array}$$ (16)

where β is the outer learning rate. The target of optimization is to learn the model parameters so that a new task can be effectively learned with a small number of gradient updates.

Compared to other meta-learning frameworks (Vinyals et al., 2016; Ravi and Larochelle 2017; Snell et al., 2017), the fine-tuning based framework, such as MAML, can be more easily applied to other optimization methods. It has been proved that fine-tuning can improve the performance of other few-shot learning methods, such as transfer learning (Sun et al., 2019). Besides the supervised learning for classification, MAML also has the potential for rapid adaptation in reinforcement learning (Gupta et al., 2018) and imitation learning for robots (Yu et al., 2018). Hence, fine-tuning based meta-learning is expected to be applied to more fields than other meta-learning approaches. There are many studies to improve the performance of MAML (Li et al., 2017; Nichol et al., 2018; Antoniou et al., 2018). Here, our work is essentially developed from MAML++(Antoniou et al., 2018), including batch normalization, layer-wise learning rate, etc.

### ROA: Rapid One-Step Adaption

Our proposed ROA method is a hybrid approach that implements offline learning in software and online learning in memristor hardware by introducing a special regularization constraint and a dynamic learning rate strategy for *in-situ* learning. Like most meta-learning methods, the learning scheme has two phases. In the pre-training phase, the network is trained from scratch in software. By learning from a group of tasks, it builds a good initial network, which can be rapidly updated for a new task. Then we map the initial network to the hardware through iterative programming with a write-verify technique to achieve high precision. This is inspired by the initial structure formed through evolution to provide a good foundation for the rapid learning ability of human beings (Jeong and Hwang 2018). The learned initial network is expected to provide the hardware with a rapid learning ability. In the task-adaptation phase, *in-situ* learning is performed in the memristor arrays. The hardware system would solve a new task with fewer data points in one update. We map the input samples to the input voltage of the memristor network and make predictions based on the output currents. Then we calculate the gradient descent by backpropagation and convert them into the number of update pulses for the specific task in the program. Finally, the hardware network is updated by one step for testing.

In our learning scheme, the initial network would be prepared in advance for the targeted tasks. Pre-training of the network can be completed before applications, such as in cloud. Therefore, for on-chip task-adaptation, only a few training samples are needed due to the previous experience gained from the pre-training. This way, the computation costs and time consumption are greatly reduced as compared to the previously reported methods. It can also be easily deployed on local or edge servers. It is worth noting that our ROA approach can also be transferred to other rapid learning strategies with fine-tuning, such as transfer learning (Pan and Yang 2010), which uses a previously trained back-bone network to quickly adapt to unseen domain.

The proposed ROA method thereby combines the advantages of offline and online training of the memristor networks. As compared to the inference-only memristor network, it achieves a similar performance due to the write-verified initial network with high precision that is implemented on hardware, and a more powerful generalization due to adaption for a new task. Meanwhile, the fast *in-situ* adaptation of ROA reduces the entanglement between the network and the hardware, thereby mitigating the accuracy loss caused by the nonlinearity and asymmetry of online learning of the memristor hardware. In the cases of limited resources, the performance of ROA is expected to exceed both online and offline methods.

To further mitigate the effects of discrete weight updates with asymmetric nonlinearity in hardware network, we develop the following two measurement methods for training:

#### Regularization Constraint

Because of the nonlinearity, the changes of memristor conductance are not proportional to the number of input pulses. As shown in Figure 2B, the conductance change close to the extreme value is more subtle than the change along the opposite direction. In other words, the precision of memristor weights depends on the state of the memristor and updating direction. In addition, compared with the simulation results, the range of weights in hardware is limited. A good initial state by limiting the weight update is expected to make the update of memristor synapses more stable. Hence, we propose a regularization constraint of the initial weights in the loss function of the meta-training as follows:

$$\begin{array}{c} \theta_{0}^{\text{*}}=\theta_{0}-\beta \nabla_{\theta }\;\underset{a}{\sum }ℒ\left(D_{test}^{\left(a\right)},\theta_{n}^{\left(a\right)}\right)-\gamma \nabla_{\theta }g\left(\theta_{0}\right), \end{array}$$ (17)

where γ is the rate of constraint. An example of the regularization constraint $g\left(\theta_{0}\right)$ is

$$\begin{array}{c} g\left(\theta_{0}\right)=\left|\theta_{0}-\rho \right|+\left|\theta_{0}+\rho \right|-2\rho , \end{array}$$ (18)

which restricts the initial weights in the range of [−ρ,ρ] . The experimental results show best performance in different tasks is achieved when ρ is around 0.5 .

#### Learning Rate Adjustment

**FIGURE 3:**
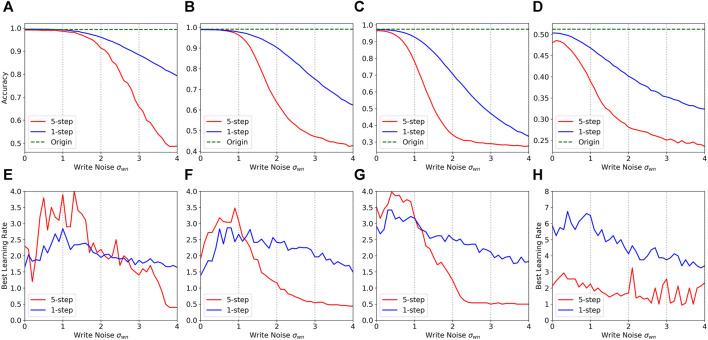
Comparison of the accuracy of 5-step update and 1-step update in different write noise and their best relative learning rate in different write noise. **(A, E)** 5way-1shot on Omniglot. **(B, F)** 10way-1shot on Omniglot. **(C, G)** 20way-1shot on Omniglot. **(D, H)** 5way-1shot on MiniImageNet.

The duration and amplitudes of pulses are fixed in the simulation. So, there is a number rounding to convert weight updating to integer numbers. However, the weight updating in the fine-tuning is usually too small to be retained in the rounding pulse number. The good news is that we can increase the inner learning rate to neutralize the influence partially. On the other hand, when the variation of devices is too large, the excessive change of weights would cause larger noises, which will adversely affect the performance. Thus, we set the inner learning rate according to the device properties, which expands at low noise levels and shrinks at high noise levels. The learning rate of the inner loop follows the setup in Antoniou et al. (2018), which is learnable for each step and each layer. We multiply the inner learning rate by a factor, called the relative learning rate. The best relative learning rate is determined by the noise level and task complexity as shown in Figure 3, which was based on experience. The details are reported in the next section.

## Experiment

In this section, we describe the implementation details of the experiments and the results of our ROA model on few-shot learning tasks. A supervised few-shot learning task can be defined as an N -way k -shot learning task. Each task provides k labeled samples in each of the N classes, which have never been trained before. There are k⋅N labeled examples in total (called a support set) in a single task. The task is to classify the unlabeled samples (called a query set) into one of these N classes. Thus, the accuracy of the random classifier in this task is 1/N . The main challenge of a few-shot learning task is that only a few samples are given. Usually, k is a small number, such as 1 or 5. This is common in applications. For example, we do not need hundreds of photos of dogs to recognize what a dog is. It requires the agent to have sufficient prior experience in performing tasks.

### Experiment Details

We conducted experiments on the Omniglot dataset (Lake et al., 2011) and miniImageNet (Ravi and Larochelle 2017). The Omniglot dataset is composed of 1,623 handwritten character classes from different alphabets. In each class, there are 20 instances in the dataset. We shuffled all classes and randomly split them into three parts: 1,150 for the training set, 50 for the validation set and 423 for testing. 15 samples are randomly picked in each class as the query set, and the rest are used as the support set according to the setting of the task. The MiniImageNet dataset is composed of 64 training classes, 12 validation classes, and 24 test classes, which is a subset of ImageNet. We evaluated our method on 5way-1shot, 10way-1shot and 20way-1shot learning tasks on the Omniglot and 5way-1shot learning task on the MiniImageNet.

The base model follows the same architecture in Antoniou et al. (2018), which has a 4-layer convolutional neural network with a 3×3 convolutions and 64 filters in each layer, followed by a batch normalization, a ReLU nonlinearity, and a 2×2 max-pooling. The last layer is a fully connected layer, which has the number of output channels corresponding to the task classes. The softmax function is applied to convert them into probability distributions over the classes. The Omniglot images are downsampled to 28×28 . For the MiniImageNet, the images are down sampled to 84×84 . The loss function is the cross-entropy error between the predicted and true classes. The training consists of 100 epochs and 500 iterations in each epoch. At the end of each epoch, we evaluated the model on the validation set. The model with the best performance on the validation set in all epochs is chosen as the final model for testing. The pre-training is done by precise computations. We assume that the process of mapping the network to memristors has enough steps to reduce the deviation of the initial network to zero. The task adaptation is *in-situ* learning on the memristor performed by simulation. In each step of the weight update, the write noises as described in Eq. 7 are added to the synapse. For comparison, the results with ideal weights are reported as the origin in the green dashed line. All experimental results were repeated four times, and then the average value was taken.

### Result Analysis

In this section, we compare ROA with offline learning and online learning. The task of the traditional learning method for memristor networks is quite different from the few-shot learning, which is usually a supervised task with a large training dataset. For offline learning, we write a well-trained network on few-shot task by using MAML to the memristor array with limited write-verify steps. More inner update steps in meta-learning are believed to help to improve performance. Here, more update steps are added on memristors as an online learning scheme for comparison.

#### Comparison With Hardware Inference

**FIGURE 4:**
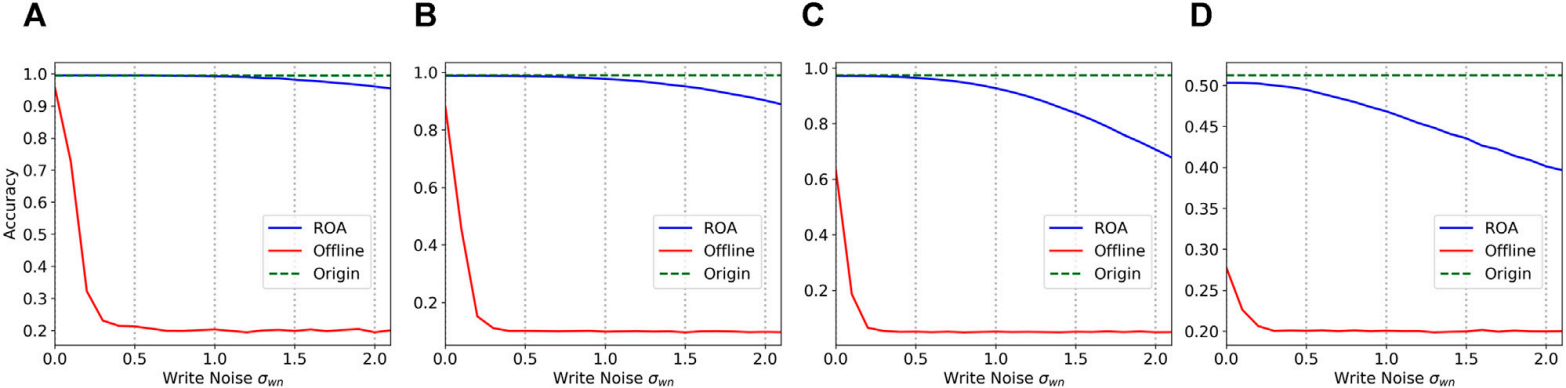
Comparison of the accuracy of 5-step update offline and ROA in different write noise. **(A)** 5way-1shot on Omniglot. (B) 10way-1shot on Omniglot. **(C)** 20way-1shot on Omniglot. **(D)** 5way-1shot on MiniImageNet.

In the comparison between offline learning and ROA, we used the same few-shot task but trained a network with ideal conditions in the simulator as the offline network. The parameters in the initial network for an offline learning follow a Gaussian distribution, while the initial network is the pre-trained network in ROA. Obviously, the offline inference can achieve results comparable to software, and there are enough of iterative writing steps to eliminate the variations in memristors. For comparison, they have the same number of update steps on hardware. Then, the trained network is mapped to the memristor array by one step writing. The ROA also updates its weights in the memristor array in one step. The tasks and training settings of the two networks are identical. The results are shown in Figure 4. We can see that due to the limitation of the iterative update steps, the offline schemes could not achieve similar performance when the write noise is zero. The accuracy rapidly drops when the noise increases. When the write noise is more than 0.3, its performance is close to random guessing. For a new unseen task, ROA only needs one update step, which is much more efficient than the offline scheme.

#### Comparison With -More Update Steps

Here, we compare the results of 1-step update and 5-step update. The weights are updated in one step with ROA while the weights are updated in five steps with online learning schemes. The initial networks are pre-trained for their schemes separately. The results are shown in Table 2, Figure 3. There is no significant discrimination between the two different conditions when the noise level is at low. The greater the noise, the lower the accuracy. Depending on the task, the accuracy of 5-step update will drop significantly when the noise reaches a certain level. The accuracy of 1-step update decreases more slowly. The more classes in the task, the smaller the decrease in the threshold of 5-step update. Their best learning rate under different write noises also has a similar declining trajectory. The intuitive explanation is that the accumulation of errors in multiple steps causes the rapid decline. Multi-step updates are not suitable for noisy hardware networks, especially in the case of complex tasks. In other words, it is not a good choice to train a network entirely on the memristor with hundreds of updates in accordance with the online learning methods.

**TABLE 2:** Comparison of the accuracy of ROA, 5-step update, ROA without constraint (ROA-WC) and fixed learning rate (ROA-FLR) in different write noise.

	$\sigma_{\text{WN}}$		0.0 (%)	1.0 (%)	2.0 (%)	4.0 (%)
		5-step	99.20	98.67	91.33	48.77
		ROA-WC	99.41	99.06	95.17	75.19
	5way-1shot	ROA-FLR	99.51	98.47	92.39	74.09
		ROA-FLR-WC	99.30	98.19	91.67	60.25
		ROA	99.53	99.21	96.39	79.57
		5-step	99.04	96.25	63.66	42.80
		ROA-WC	99.00	97.55	87.23	52.06
	10way-1shot	ROA-FLR	98.79	96.02	82.74	56.96
Omniglot		ROA-FLR-WC	98.93	95.13	77.14	35.09
		ROA	98.87	97.71	90.25	62.39
		5-step	96.58	78.07	34.31	27.65
		ROA-WC	97.85	91.90	66.83	32.15
	20way-1shot	ROA-FLR	96.95	85.41	55.12	28.22
		ROA-FLR-WC	97.77	86.67	57.68	21.24
		ROA	97.21	92.74	70.70	33.39
	20way-1shot wider	ROA-WC	97.97	89.60	64.87	32.57
		ROA	97.68	92.25	72.91	40.99
		5-step	48.04	38.94	28.09	23.56
		ROA-WC	49.42	44.46	37.66	30.64
MiniImageNet	5way-1shot	ROA-FLR	46.26	39.46	33.93	29.81
		ROA-FLR-WC	42.64	36.53	31.37	27.56
		ROA	50.33	46.84	40.13	32.38

### Ablation Study

**FIGURE 5:**
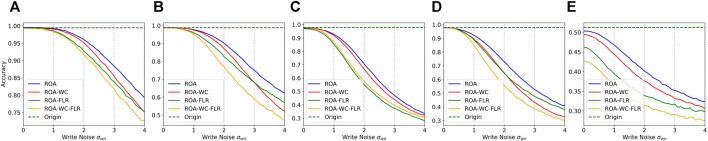
Comparison of the accuracy of ROA, ROA without constraint (ROA-WC), no adjustment on learning rate (ROA-FLR) and baseline (ROA-WC-FLR) in different write noise. **(A)** 5way-1shot on Omniglot. **(B)** 10way-1shot on Omniglot. **(C)** 20way-1shot on Omniglot. **(D)** 20way-1shot with the wider network on Omniglot. **(E)** 5way-1shot on MiniImageNet.

In this section, we further conduct several ablation experiments to demonstrate the functionality of our method. We compare ROA with ROA without constraint (ROA-WC), no adjustment on learning rate (ROA-FLR) and MAML baseline (ROA-WC-FLR) in different write noise, respectively. The results are shown in Figure 5.

#### Impact of Constraint

We investigated the impact of constraints on the accuracy of one-shot tasks in different ways. The comparisons of accuracy are shown in Figure 5, Table 2. The constraint used is Eq. 18 with ρ=0.5 . When ρ=1 , the range of constraint is the same as the range of synaptic weights. We treat it as ROA without the constraint. As shown in Figures 5A,B,E, the constraint improves the accuracy by more than 3% when ${\text{σ}}_{\text{WN}}$ increases to 4. But the accuracy of these two approaches is similar in the task of 20-way 1-shot in Figure 5C. We further evaluated the wider network with 128 filters for this task in Figure 5D. It shows that the accuracy of ROA is improved by about 2% for wider networks, but the accuracy of ROA without constraint is even slightly reduced in a high-noise setting. The results suggest that the network capacity can help improve performance only when the network is constrained under noises. The constraint is only effective under noises, otherwise there is no obvious improvement under ideal conditions.

#### Impact of Learning Rate Adjustment

**FIGURE 6:**
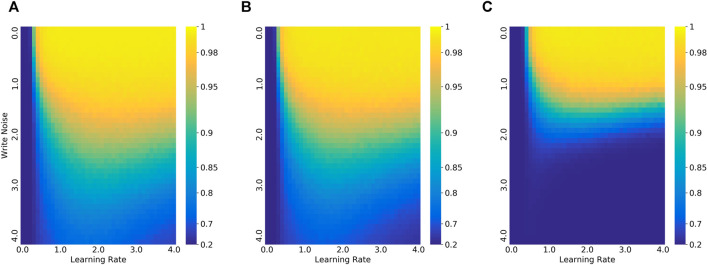
The impact of write noise and learning rate on 5way-1shot Omniglot task. **(A)** ROA. **(B)** ROA without constraint (ROA-WC). **(C)** 5-step update ROA.

The best learning rate is determined by the validation set in the experiment. Figure 3 plots the learning rates against write noises. As the noise increases, the best learning rate increases slightly at the beginning, ${\text{σ}}_{\text{WN}}<1$ , and then decreases as the accuracy decreases. The best learning rate would drop below 1 (about 0.5). The unexpected effect is too noisy for the network. Furthermore, we also compared the results between the original learning rate (keep at 1) and the best learning rate in Figure 5, Table 2 to illustrate the superiority of our method. Similar to the previous item, their difference at low noise is very small, and increases with the increase in write noise, especially in more complex tasks. The accuracies of 5way-1shot, 10way-1shot and 20way-1shot tasks on Omniglot and 5way-1shot tasks on MiniImageNet are improved by about 4, 7, 15, and 5%, respectively, when the ${\text{σ}}_{WN}$ is greater than 2. The impact of learning rate and write noise in 5way-1shot Omniglot tasks is plotted in a heatmap as shown in Figure 6. The result suggests that the best learning rate is about 2 for Omniglot and 4 for MiniImageNet under acceptable noise.

## Discussion and Conclusion

In this work, we developed a bi-level meta-learning scheme, ROA, for memristor neural networks. It is a hybrid approach that combines online learning and offline learning, which can effectively alleviate the impact of the non-ideal properties of memristors through one update step. A simulator was built based on the parameters extracted from our memristor devices and evaluated using the Omniglot dataset and MiniImageNet dataset. Our experimental results demonstrate that the ROA method can significantly improve data efficiency and training speed, thereby achieving better performance than multi-step adaption and offline learning under similar conditions. In addition, our method shows a strong robustness to noise, which facilitates the real-world applications of memristor networks. The results suggest that, with the proposed, the memristors are suitable as an accelerator for rapid learning hardware rather than just a hardware inference or *in-situ* learning with massive updates.

Moreover, memristor networks and the ROA method can benefit from each other. The rapid adaption of ROA requires that the weights in the neuromorphic hardware have on-chip plasticity, which can be easily achieved by memristor networks. On the other hand, the one-step adaption allows the hardware network to extricate the weight update from the non-ideal properties of memristors, thereby reducing the accuracy loss in the mapping process. Collectively, memristors are very suitable for accelerators to achieve learning-to-learn capability. Our ROA scheme can improve the performance of memristors, and facilitate broad applications in neuromorphic architecture. The rapid adaptation process could be implemented in a local mode without the support of cloud servers, indicating a low adaptation latency. Hence, the users’ personal data do not need to be uploaded to server, ensuring privacy and security. Furthermore, the flexible learning scheme can benefit hardware neural networks to handle uncertain environments and individual demands.

## Data Availability

The original contributions presented in the study are included in the article/Supplementary Material, further inquiries can be directed to the corresponding author.
